# Centipede (Chilopoda) richness and diversity in the Bug River valley (Eastern Poland)

**DOI:** 10.3897/zookeys.510.8763

**Published:** 2015-06-30

**Authors:** Małgorzata Leśniewska, Piotr Jastrzębski, Marzena Stańska, Izabela Hajdamowicz

**Affiliations:** 1 Department of General Zoology, Adam Mickiewicz University ul. Umultowska 89, 61-614 Poznań, Poland; 2 “Natura” Ecology Research Laboratory Marek Wierzba, ul. Kubusia Puchatka 78, Żabokliki 08-110 Siedlce, Poland; 3 Siedlce University of Natural Science and Humanities, Faculty of Natural Sciences, Department of Zoology, ul. B. Prusa 12, 08-110 Siedlce, Poland

**Keywords:** Big river valley, centipedes, species richness, habitat preferences

## Abstract

The main aim of the survey was to describe the diversity and richness of Chilopoda in the selected area of the Bug River valley. The study sites were located in two regions differing in the shape of the valley, the presence of thermophilous habitats and the size of riparian forests. Pitfall traps were used as a sampling method. As a result, 444 specimens belonging to 12 centipede species of two orders – Geophilomorpha (four species) and Lithobiomorpha (eight species) were caught. Lithobius (Monotarsobius) curtipes C.L.Koch, 1847, *Pachymerium ferrugineum* (C.L.Koch, 1835), Lamyctes (Lamyctes) emarginatus (Newport, 1844) and Lithobius (Monotarsobius) dudichi Loksa, 1947 were the most common and the most numerous species. Of particular note is *Lithobius dudichi* found in Poland for the first time and previously known based on a single specimen. Two to 10 Chilopoda species were found in each habitat under investigation. The greatest species richness was found in thermophilous thickets (10 species), sandy grasslands (eight), xerothermic grasslands (eight) and mesic meadows (six). The fewest number of species (two) was found in rushes at oxbows and in wet meadows. We found differences in the species composition and the number of Chilopoda between the lower (102 specimens, six species) and the middle (324 specimens, 11 species) section of the river valley. Our results confirm the need to protect xerothermic habitats, unique almost throughout entire Central Europe, which due to their distribution and their small area covered are fairly easily subject to the process of destruction.

## Introduction

Habitats in valleys of European rivers are relatively poorly known in terms of species diversity, habitat selection and the dynamics of Chilopoda communities. The very few studies in this field include, for instance, Zerm (1997a,b, 1999). In Poland, such studies have not been conducted so far.

Centipedes from river valleys have been studied mainly in the context of changes in the communities as a result of seasonal flooding (Zulka 1991, Pižl and Tajovský 1998, Tajovský 1999, Tuf 2000, 2003, Tufová and Tuf 2005, Marx et al. 2009), and in the context of life strategies enabling survival in periodically flooded habitats (Adis et al. 1996, Adis and Junk 2002). Xerothermic environments (often naturally occurring in the river valleys) have rarely been the subject of research on Chilopoda (Voigtländer 2003).

River valleys, especially the natural ones, only slightly changed – unregulated, are extremely valuable areas with habitats found more and more rarely, which already start to disappear across the continent. Studies on these habitats provides an opportunity not only to learn about the biodiversity but also to develop appropriate management and protection schemes. The Bug River is one of the few rivers of such a size in Europe, which still remain almost unregulated (Dombrowski et al. 2002). Its length in Poland amounts to 587 km.

The aim of this study is to describe the diversity and richness of Chilopoda in the selected area of the Bug valley through:

- determination of the species composition and structure of Chilopoda communities in different habitats of the Bug valley;
- distinction of the most numerous species in a given habitat and those that are most flexible in many environments;
- determination of the habitat preferences of centipede species in the studied areas.

## Materials and methods

Research in the Bug river valley was conducted since March to November in 2007 and 2008.

## Study area

**Figure 1.**
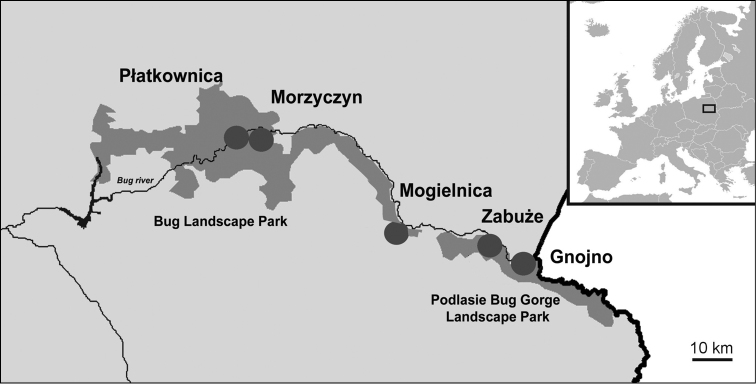
Location of the study area in Poland. Study area. Morzyczyn, Płatkownica – lower section of the Bug valley: Mogielnica, Zabuże, Gnojno – central section of the Bug valley.

The study sites were located in two regions differing in the shape of the valley, the presence of thermophilous habitats and the size of riparian forests (Figure 1):

1. In the middle section of the river, as it meanders and cuts through glacial uplands at the depth of 30 m creating a unique landscape in Europe, where the characteristic feature is the presence of thermophilous habitats and well-preserved riparian forests (near localities: Gnojno, Zabuże in the protected area ‘Podlasie Bug Gorge Landscape Park’, and near locality Mogielnica in the protected area ‘Bug Landscape Park’);
2. In the lower course of the river, where the valley is much wider (it stretches up to several kilometers wide) with an overgrown flood terrace at its bottom. In this section, the Bug river slowly meanders and sometimes changes its course (near localities: Morzyczyn, Płatkownica, in the protected area ‘Bug Landscape Park’).

In the Bug valley we can come across habitats that vary in terms of moisture content and structure – two important parameters from the point of view of Chilopoda biology. On the side of the river – from flooded and very humid habitats through medium moist ones to grasslands. Riparian forests within the floodplain are closest to the river, then there are meadows of lower flooded terrace – submerged during river floods. Mesic meadows are found in higher terraces, while slopes of terraces feature xerothermic grasslands, and thermophilous thickets. Sandy grasslands and rushes at oxbows are located in the mosaic of meadows, sometimes closer to the river bed, and sometimes closer to the edge of the valley (Głowacki et al. 2002). In terms of the structure, according to the classification by Voigtländer (2005) one can distinguish high (riparian forests), middle (thermophilous thickets, rushes at oxbows) and low (meadows, grasslands) vegetation cover.

Below, the data about the habitat (along with an abbreviation used throughout the study), coordinates, phytocoenosis, location, substrate, at every site are given.

The coordinates of the sites were determined using a GPS device by Garmin.

**Sandy grassland (sgr)**:

1) Gnojno, sandy grassland; 52°28'13"N, 23°13'64"E; *Diantho*-*Armerietum*; raised, flat flooded terrace, several meters from the clearly visible edge of the meadow terrace; desiccated river alluvial soils;

2) Morzyczyn, sandy grassland; 52°68'43"N, 21°91'99"E; *Sclerantho*-*Herniarietum glabre*; the community inhabits the upper flooded terrace in the vicinity of the Bug riverbed; desiccated river alluvial soils;

3) Płatkownica, sandy grassland; 52°69'02"N, 21°84'53"E; *Diantho*-*Armerietum*; the phytocenosis occupies the raised, flat flooded terrace at the foot of the southern slopes of the flood embankment, within the base of the Bug valley cut off from the inundation area; desiccated river alluvial soils.

**Xerothermic grassland (xgr)**:

4) Gnojno, xerothermic grassland; 52°27'59"N, 23°13'88"E; impoverished form of the *Adonido*-*Brachypodietum arrhenatheretosum*; slope of the upper terrace, southern exposure and inclination of approx. 30°; proper parendzina;

5) Mogielnica, xerothermic grassland; 52°40'08"N, 22°57'04"E; *Adonido*-*Brachypodietum*; slope of the upper terrace, eastern exposure and inclination of approx. 30°; proper pararendzina;

6) Morzyczyn, xerothermic grassland; 52°68'43"N, 21°91'50"E; *Tunico*-*Poetum compresse*; the analyzed patch of the phytocoenosis evolved in an anthropogenic habitat, with a slightly alkaline pH. It is the southern slope of the flood embankment with an inclination of about 30°; anthropogenic pararendzinas;

7) Płatkownica, xerothermic grassland; 52°69'00"N, 21°84'43"E; grassland with *Carex praecox* of the *Agropyretea intermedio-repentis* class; southern slope of the flood embankment with an inclination of about 35 degrees; anthropogenic pararendzinas.

**Rushes at oxbows (rux)**:

8) Gnojno, rushes at oxbows; 52°28'20"N, 23°13'64"E; *Phalaridetum arundinaceae*; periphery of the Bug river, between club-rushes – on the water side – and flooded grasslands (*Agropyro*-*Rumicion*) on the mainland side; alluvial soils;

9) Mogielnica, rushes at oxbows; 52°40'06"N, 22°57'40"E; *Glycerietum maximae*; in the final, very shallow stretch of the Bug river, surrounded by tall herb communities and meadow communities on the land side; alluvial soils;

10) Morzyczyn, rushes at oxbows; 52°68'17"N, 21°91'24"E; *Glycerietum maximae*; located in a very shallow, bank stretch of the Bug river, surrounded by meadow communities on the land side and by *Caricetum gracilis* reed on the water surface side; alluvial soils;

11) Płatkownica, rushes at oxbows; 52°69'21"N, 21°84'54"E; *Glycerietum maximae*; located on the edge of the shallow old riverbed of the Bug river; alluvial soils.

**Thermophilous thickets (tht)**:

12) Gnojno, thermophilous thickets; 52°28'19"N, 23°13'47"E; *Rhamno*-*Cornetum sanguinei*; on the edge of the Bug valley. The phytocoenosis habitat is a moraine slope with eastern exposure and inclination of approx. 30°; leached brown soils;

13) Mogielnica, thermophilous thickets; 52°40'02"N, 22°57'08"E; *Rhamno*-*Cornetum sanguinei*; on the edge of the Bug valley. The phytocoenosis habitat is a moraine slope with eastern exposure and inclination of approx. 30°; brown soils.

**Mesic meadow (mm)**:

14) Zabuże, mesic meadow; 52°33'32"N, 23°00'00"E; *Poo*-*Festucetum*; raised upper flooded terraces of the Bug valley; river alluvial soils;

15) Mogielnica, mesic meadow; 52°40'08"N, 22°57'22"E; *Poo*-*Festucetum*; raised upper flooded terrace of the Bug valley; river alluvial soils;

16) Morzyczyn, mesic meadow; 52°68'51"N, 21°91'54"E; *Poo*-*Festucetum*; raised upper flooded terraces of the Bug valley; river alluvial soils;

17) Płatkownica, mesic meadow; 52°69'06"N, 21°84'53"E; *Poo*-*Festucetum*; raised upper flooded terraces of the Bug valley; river alluvial soils.

**Riparian forest (rfo)**:

18) Zabuże, riparian forest; 52°33'33"N, 23°00'86"E; *Salicetum albae-fragilis*; N slope of the low flooded terrace in the patch of the riparian willow; alluvial processes accumulate coarse-grained material of sand and river sediments. During periods of low water the initial alluvial soil may undergo significant desiccation;

19) Mogielnica, riparian forest; 52°40'32"N, 22°57'21"E; *Salicetum albae-fragilis*; the flooded terrace; proper alluvial soil;

20) Morzyczyn, riparian forest; 52°69'05"N, 21°91'36"E; *Salicetum albae-fragilis*; slightly elevated riverbed bank, within the flooded terrace; initial alluvial soil;

21) Płatkownica, riparian forest; 52°69'19"N, 21°84'06"E; *Salicetum albae-fragilis*; flooded terrace; proper alluvial soils.

**Wet meadow (wm)**:

22) Zabuże, wet meadow; 52°33'23"N, 22°99'95"E; *Violo*-*Cnidietum*; upper flooded terrace of the Bug valley, submerged regularly during the floods of the river; river alluvial soils;

23) Morzyczyn, wet meadow; 52°68'84"N, 21°91'61"E; a meadow with *Carex praecox* and *Poa angustifolia* (formed from the disturbed *Cnidion* meadow); lower flooded terrace, currently submerged only occasionally during high floods of the river; desiccated river alluvial soils;

24) Płatkownica, wet meadow; 52°69'16"N, 21°84'42"E; *Violo*-*Cnidietum*; upper flooded terrace of the Bug valley, submerged regularly during the floods of the river; river alluvial soils.

Pitfall traps were used as a sampling method. An aqueous solution of propylene glycol (about 50%), containing a few drops of a detergent per 1 liter to reduce the surface tension of the fluid, was used as a preservation liquid. In each of the sites ten pitfall traps were placed in one straight line, at a distance of two meters one from another. The beginning of the trapping period was in the middle of March and the end was in the middle of November. The traps were replaced every two weeks.

The material analyzed in the current work was obtained during studies related to different groups of arthropods – including primarily spiders, carabids, diplopods and butterflies – under the project titled “The diversity of habitats and the biological diversity of selected groups of Arthropoda in the Bug valley” (Oleszczuk et al. 2011, Jastrzębski 2012, Hajdamowicz et al. 2014). Since Chilopoda were not taken into account during the planning of the study, the specificity of this group of animals was not accounted for in the applied methodology.

In this work, standard methods and analysis indicators were applied: The Shannon-Weaver diversity index (H), Pielou’s measure of species evenness (J), Morisita index values as modified by Horn, the cluster analysis – distance/similarity measure Bray and Curtis; cluster method: nearest neighbor.

## Results

### Species

444 specimens belonging to 12 centipede species of two orders – Geophilomorpha (four species) and Lithobiomorpha (eight species) were caught (Table 1).

**Table 1.** List, the number (N), dominance (D%) of Chilopoda species found in the Bug valley; Values of the index of species diversity by Shannon-Weaver (H’), the highest (H max) value of the Shannon-Weaver index for individual habitats and values of Pielou’s measure of species evenness (J); *– species new for Poland; rfo – riparian forests, rux – rushes at oxbows, sgr – sandy grasslands, xgre – xerothermic grasslands on embankments, tht – thermophilous thickets, mm – mesic meadows, wm – wet meadows, xgr – xerothermic grasslands. Ecological and zoogeografical classification of species – Af – African, Austras – Australasian, e – eurytopic; E – European; f – forest; H – Holarctic; i – introduced; NA – North American; Naf – North African; s – synanthropic; WP – West Palearctic; ? – unknown.

	Species/habitats	Ecol. and zoog. classif.	thermophilous thickets (tht)		sandy grassland (sgr)		xerothermic grassland (xgr)		mesic meadow (mm)		riparian forest (rfo)		rushes at oxbows (rux)		wet meadow (wm)		no data	total		number of habitats
			N	D	N	D	N	D	N	D	N	D	N	D	N	D	N	N	D	
1	Lamyctes (Lamyctes) emarginatus (Newport, 1844)	e, Austras, E, Am, Af			1	2.4	1	1.6	15	17.6	12	8.6	20	95.2	11	84.6	1	61	14.3	6
2	Lithobius (Monotarsobius) curtipes C.L.Koch,1847	f, P	4	9.8	1	2.4	11	18	5	5.9	122	87.8	1	4.8			14	158	37.0	7
3	Lithobius (Monotarsobius) dudichi Loksa, 1947 *	?	5	12.2	22	52.4	20	32.8	9	10.6	5	3.6					2	63	14.8	5
4	Lithobius (Lithobius) erythrocephalus erythrocephalus C.L.Koch,1847	e, E	1	2.4	1	2.4												2	0.5	2
5	Lithobius (Lithobius) forficatus forficatus (Linnaeus, 1758)	e, WP	4	9.8	1	2.4	1	1.6									1	7	1.6	3
6	Lithobius (Lithobius) melanops melanops Newport, 1845	e, s, E	1	2.4														1	0.2	1
7	Lithobius (Lithobius) mutabilis mutabilis L.Koch, 1862	f, E	17	41.5	1	2.4	2	3.3										20	4.7	3
8	Lithobius (Lithobius) tenebrosus tenebrosus Meinert, 1872	f, E															1	1	0.2	
	*Lithobius* species		5	-	1	-	5	-	1	-	5	-						17	-	
9	*Geophilus proximus* C.L.Koch,1847	e, E, iNA	2	4.9	1	2.4	1	1.6	3	3.5								7	1.6	4
10	*Pachymerium ferrugineum* (C.L. Koch, 1835)	e, H	1	2.4	14	33.3	22	36.1	49	57.6					2	15.4	5	93	21.8	6
11	*Schendyla nemorensis* (C.L. Koch, 1837)	e, s, E, Naf, iNA	4	9.8			3	4.9	4	4.7							1	12	2.8	3
12	*Strigamia acuminata* (Leach, 1815)		2	4.9														2	0.5	1
	number of specimens		46		43		66		86		144		21		13		25	444		8
	number of species		10		8		8		6		3		2		2			12		
	number of sites		2		3		4		4		4		4		3					
	H’		0.81		0.54		0.65		0.56		0.19		0.08		0.19					
	H max		1.0		0.90		0.90		0.78		0.48		0.30		0.30					
	J		81.2		59.6		72.4		72.0		40.6		27.6		61.9					

In the area under investigation, the following four centipede species were most numerous and most common:

1. Lithobius (Monotarsobius) curtipes – present in six habitats, with the highest number in riparian forests (77% of specimens);
2. *Pachymerium ferrugineum* – found in five habitats, most abundant in the mesic meadows (53% of specimens);
3. Lamyctes (Lamyctes) emarginatus – caught in six habitats, most numerous in very wet habitats – in rushes at oxbows (33% of specimens), in the wet meadows (25%) and in riparian forests (20%);
4. Lithobius (Monotarsobius) dudichi – present in five habitats, prevalent in sandy grasslands (35% of specimens) and in xerothermic grasslands (32%).

These species constitute 88% of all centipedes caught during the study, thus establishing themselves as the most typical ones of almost all habitats of the study area. It is only in thermophilous thickets that a species from outside this group of four dominates – Lithobius (Lithobius) mutabilis (Table 1).

**Figure 2.**
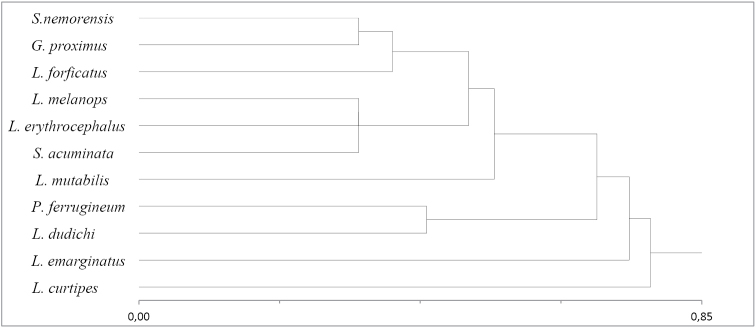
Similarity of species (distance/similarity measure Bray and Curtis; cluster method: nearest neighbor). Species nomenclature in Table 1.

Interspecies occurrence similarity (Figure 2) [the analysis does not include Lithobius (Lithobius) tenebrosus, as regrettably the information about the site on the label describing the specimen was completely obscured].

Three distinct groups can be identified in the dendrogram. The first group includes species associated with dry habitats, which are rarely found in the Bug valley (*Schendyla nemorensis*, *Geophilus proximus* and *Lithobius forficatus* related to these species). The second cluster consists of species occurring mainly in thermophilous thickets (*Lithobius melanops*, *Lithobius erythrocephalus* and *Strigamia acuminata*). *Lithobius mutabilis* – which is dominant in thermophilous thickets, but also found in sandy and xerothermic grasslands, is related to these two groups. The last cluster consists of numerous and frequently found species of *Pachymerium ferrugineum* and *Lithobius dudichi*, which occur together in four habitats. In sandy grasslands, xerothermic grasslands and in thermophilous thickets their number is similar. The most distinctive species are *Lithobius curtipes* – found most frequently in the Bug valley and reported from the great number of habitats, although clearly predominant in riparian forests and *Lamyctes emarginatus* – also caught in many habitats, although predominantly in wetlands and in regularly flooded areas.

In terms of ecology and zoogeography European eurytopic species prevail (Table 1).

Chilopoda were collected throughout all the months of the study, although one can observe certain tendencies in particular species – especially with regard to four, most frequent species. *Lithobius curtipes* was active throughout all the months, though predominantly in October and November. *Lamyctes emarginatus* occurred from June to November, while it was most numerous in September. *Pachymerium ferrugineum* was reported from April until November, especially in May and June, but also in August and September. Finally, *Lithobius dudichi* was most active in May and November.

### Habitats

Two to ten Chilopoda species were found in each habitat under investigation. The greatest species richness was found in thermophilous thickets (ten species), sandy grasslands (eight species), xerothermic grasslands (eight species) and mesic meadows (six species). The fewest number of species was found in rushes of reed mannagrass (*Glyceria maxima*) and in wet meadows (two species at each location) (Table 1).

The greatest number of specimens was found in the following habitats: riparian forests, mesic meadows and xerothermic grasslands (Table 1).

In the majority of habitats one species was overwhelmingly dominant (from 41.5% to 95.2%). In rushes at oxbows and in the wet meadows it was *Lamyctes emarginatus*, in riparian forests *Lamyctes curtipes*, in thermophilous thickets *Lamyctes mutabilis*, in sandy grasslands *Lithobius dudichi*, and in the mesic meadows *Pachymerium ferrugineum*. It was only in xerothermic grasslands that two species co-dominated (*Lithobius dudichi* and *Pachymerium ferrugineum*) (Table 1).

The Shannon-Weaver diversity index (H) and Pielou’s measure of species evenness (J) reached their highest values in thermophilous thickets, xerothermic grasslands, mesic meadows, while the lowest values – in rushes at oxbows and riparian forests (Table 1).

**Figure 3.**
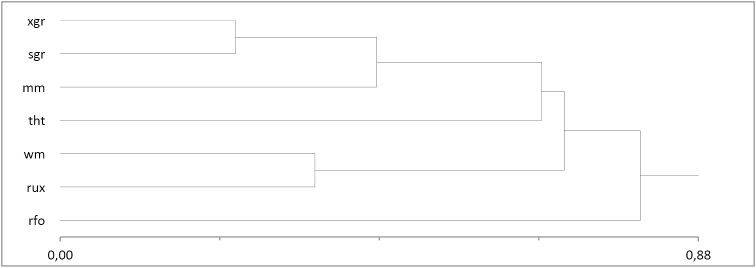
Dendrogram of the similarities of centipede composition in different habitats (distance/similarity measure – Bray and Curtis, cluster method –nearest neighbor) For designations see Table 1.

The cluster analysis conducted on the basis of the species composition and dominance structure demonstrated the greatest similarity between communities of warm and dry habitats on the one hand, and wet and flooded – on the other (Figure 3, Table 2).

**Table 2.** Similarity of dominance structures – Morisita index values as modified by Horn. For designations see Table 1.

tht	1						
sgr	0.29	1					
xgr	0.35	0.90	1				
rfo	0.18	0.07	0.33	1			
rux	0.01	0.04	0.04	0.15	1		
wm	0.01	0.13	0.14	0.10	0.98	1	
mm	0.13	0.66	0.79	0.12	0.26	0.42	1
	tht	sgr	xgr	rfo	rux	wm	mm

We note differences in the species composition and the number of Chilopoda between the lower (102 specimens from 6 species) and the middle section of the river valley (324 specimens from 11 species). This result reflects the differences in the structure and the vegetation of the two regions of the Bug valley – especially the presence of thermophilous habitats in the middle section of the valley.

## Discussion

Although several studies from European river valley areas have been conducted, this habitat is still poorly explored in terms of Chilopoda. Meanwhile natural and seminatural habitats associated with valleys of big rivers are already disappearing throughout the continent (Głowacki et al. 2002).

This study is based on the materials obtained in studies on groups of arthropods other than Chilopoda. This should explain the applied methodology, which does not take into account the specificity of Chilopoda. This undoubtedly affected the results. Pitfall traps limited the set of species to active epigeic forms. The expansion of the method to include quantitative soil samples and direct qualitative capture would contribute to a more complete picture of this group of Chilopoda.

The data about the preferences of the majority of species with regard to the habitats was confirmed in the investigated area. The wettest and flooded habitats feature the lowest number of Chilopoda species. This is understandable, as the colonization of periodically flooded habitats requires special adaptations from animals in morphology, physiology or life cycle (Adis and Junk 2002, Zulka 1999, Voigtländer 2011). The species that definitely prevails in rushes at oxbows is the introduced *Lamyctes emarginatus* – a parthenogenetic species, commonly found not only in floodplains, but also in disturbed upland areas (Eason 1964, Dunger and Voigtländer 1990, Zulka 1991, Adis and Junk 2002, Leśniewska 2004). It is known as a one-year ‘autumn species’ (Barber and Keay 1988, Zulka 1991). *Lamyctes emarginatus* has a preference for humid and very wet habitats with low vegetation cover (Voigtländer 2005). In this species flood resistance of inactive stages (eggs) was found (Zulka 1991). Eggs survive inundation between winter and early spring in dormancy. Immatures hatch shortly thereafter and reach maturity already 6–12 weeks later, in contrast to most lithobiomorph species which need a few years until they are mature. *Lamyctes emarginatus* actively dives, walks under water and hunts for prey near the water surface (Adis and Junk 2002).

As noted by Zulka (1999) and Voigtländer (2011), the one-year life cycle of *Lamyctes emarginatus* is a strategy that allows this species to populate the same habitats as *Lamyctes curtipes* – a species of a similar body size and probably very similar ecological requirements. Adult specimens of the annual species *Lamyctes emarginatus* appear in the environment in the summer and fall, when there is enough food to suffice for perennial species, such as *Lamyctes curtipes*.

The riparian forest habitat is dominated by *Lamyctes curtipes* – a species that prefers wet and humid habitats with high vegetation cover. This species, was found alive after 34 days of inundation (Adis and Junk 2002). In Central Europe it inhabits primarily riparian forests, alder swamp forests, river and brook sides, wet meadows with flooding and more rarely humid deciduous forests (Voigtländer 2005). In the investigated area a small number of the specimens of this species was also collected from dry and xerothermic habitats (Table 1). It can be assumed that these specimens have only immigrated from surrounding habitats, and they do not form stable populations in these habitats.

In the Bug valley greater species diversity is found in sandy and xerothermic grasslands, where *Pachymerium ferrugineum* and *Lithobius dudichi* prevail. *Pachymerium ferrugineum* is one of the most widely spread Chilopoda species. It is found throughout the entire Holarctic. This species appears to be less sensitive to changes in moisture and temperature than other species of Chilopoda and it is often found in the littoral zone, and also in open environments – in meadows, grasslands, cultivated fields, on open rocks etc. (Palmén and Rantala 1954, Eason 1964, Barber 2009). Suomalainen (1939) reports that *Pachymerium ferrugineum* can survive under water up to 178 days, which explains its presence on numerous islands, for example in the Faroe Is., Azores, Madeira, the Canary Is. In the Bug valley *Pachymerium ferrugineum* is mainly found in habitats with a low vegetation cover and featuring various levels of moisture – both in flooded terraces and grassland slopes, which confirms the preferences of the species reported from other areas.

Most species in the Bug river valley inhabit thermophilous thickets – an environment that is already similar to forest habitats. This is clearly manifested by the composition of Chilopoda community, in which we can find typically forest-dwellers – such as *Lithobius mutabilis* or *Strigamia acuminata*.

As one of the most important results obtained in the present study is the reported high number of specimens of *Lithobius dudichi*, the species new to the Polish fauna. This species has so far been known to science only on the basis of one reported specimen (Loksa 1947, Matic 1966). This finding shows that river valley habitats are still poorly investigated and one should pay more attention to them in order to gain more comprehensive knowledge about the diversity of Chilopoda. Loksa (1947) reports *Lithobius dudichi* from one site in Romania. The fact that these two sites are so far away from each other may suggest that the range of the species is probably very large. This may be the Eastern European or the Pontic range. The Bug river gorge is the gateway for migratory species from the widely interpreted East-Ukrainian and Russian steppes and the Pontic region (along the eastern Carpathian belt and further north). These migrations have enriched Polish fauna in south-eastern species: for exemple xerothermophilous weevils (Wanat and Gosik 2003), thermophilous and higrophilous spiders (Hajdamowicz and Jastrzębski 2007, Oleszczuk et al. 2011, Hajdamowicz et al. 2014) and diplopods (Jastrzębski 2012).

Our results confirm the need to protect xerothermic habitats, unique almost throughout entire Central Europe, which due to their dispersion and their small area covered are fairly easily subject to the process of destruction. These environments are refuges for rare species of animals – including centipedes, as our research shows. *Lithobius dudichi* presumably belongs to the relict xerothermic species of steppe provenance and is presumably in danger of extinction.

The results from our research in the Bug Valley also show that centipedes are a valuable indicator group for the assessment of habitat conditions. The information about the species composition of Chilopoda communities, the dominance structure and their dynamics may thus be useful in characterizing specific location types.
